# Reading and Writing Abilities in Students with Mild Nonspecific Intellectual Disability: A Multivariate Examination of Literacy and Cognitive Processing Abilities

**DOI:** 10.3390/jintelligence13120161

**Published:** 2025-12-08

**Authors:** Urszula Sajewicz-Radtke, Ariadna Beata Łada-Maśko, Paweł Jurek, Michał Olech, Bartosz Mikołaj Radtke

**Affiliations:** 1 Laboratory of Psychological and Educational Tests, Ul., Czarnieckiego 5A/1, 80-239 Gdańsk, Poland; sajewicz-radtke@pracowniatestow.pl; 2 Institute of Psychology, University of Gdansk, Ul. Bażyńskiego 8, 80-309 Gdańsk, Poland; ariadna.lada@ug.edu.pl (A.B.Ł.-M.); pawel.jurek@ug.edu.pl (P.J.); 3 Department of Psychology, Medical University of Gdańsk, Ul. M. Skłodowskiej-Curie 3a, 80-210 Gdańsk, Poland; michal.olech@gumed.edu.pl

**Keywords:** disorders of intellectual development, literacy, cognitive profiles, phonological processing, decoding

## Abstract

Individuals with mild nonspecific intellectual disability (NSID) often exhibit delayed literacy development. Unfortunately, how cognitive–linguistic processing profiles influence literacy in this population lacks clarity. This study investigated literacy development in this population, considering the cognitive–linguistic mechanisms. The Specialist Battery for the Diagnosis of Cognitive Abilities and School Skills was used to assess cognitive–linguistic abilities and literacy-related skills in 122 participants. Fuzzy C-means clustering was used to identify processing profiles. Developmental age equivalents in literacy were estimated using local regression models and matched comparisons with typically developing peers. Two cognitive–linguistic profiles emerged: globally weaker and moderately developed. Those with NSID performed significantly lower than their peers in all domains. Their literacy skills aligned with those of children 2–4 years younger, and plateaued after age 15. Cognitive–linguistic heterogeneity in students with NSID should guide targeted literacy interventions. The findings inform ICD-11 educational expectations for individuals with mild NSID.

## 1. Introduction

The ability to read influences school and employment successes and general quality of life. Thus, it is concerning that students with intellectual disability frequently leave school owing to limited reading skills (Wei et al. 2011). While most children with intellectual disability acquire some level of reading proficiency, the degree of success varies considerably between individuals (Koritsas and Iacono 2011; Wei et al. 2011). Therefore, accurately assessing their literacy achievements is essential to inform effective intervention development (Allor et al. 2014; Ratz and Lenhard 2013).

Previous research has often concentrated on individuals with specific aetiologies of intellectual disability, particularly Down syndrome and Williams syndrome (Di Blasi et al. 2018; Lettington and Van Herwegen 2024; Næss et al. 2012). Other studies have combined individuals with intellectual disability and those with borderline intellectual functioning (IQ 70–84) (van Tilborg et al. 2014; van Wingerden et al. 2017) or have included individuals with different severity levels of intellectual disability (Ratz and Lenhard 2013). Such methodological choices may obscure meaningful patterns in literacy development and make tailored intervention design difficult.

Recent systematic reviews (e.g., Nilsson et al. 2025) have synthesised the existing evidence of the cognitive and language abilities associated with reading in individuals with intellectual disability. These reviews underscore the importance of examining not only phonological skills and processing speed but also oral language abilities, such as vocabulary knowledge and syntactic comprehension, as key predictors of reading proficiency.

Building upon the Carroll–Horn–Cattell (CHC) theory, a comprehensive framework for understanding cognitive functioning beyond a singular IQ score (McGrew and Evans 2023; McGrew 2009), our research addresses the complexities of assessing literacy skills in individuals with mild nonspecific intellectual disability. This aligns with the International Classification of Diseases (ICD-11) diagnostic approach, which highlights the inadequacy of IQ as a sole indicator of cognitive functioning and stresses the need to evaluate broader domains, including literacy. Understanding both literacy levels and underlying cognitive and linguistic mechanisms is crucial for developing effective, individualised interventions (Nilsson et al. 2025). In line with ICD-11 terminology, we use the term nonspecific intellectual disability (NSID) to refer, in the context of this study, to disorders of intellectual development of mild severity for which no specific biomedical aetiology has been identified. This includes students with clinically diagnosed mild intellectual disability who do not present with known genetic syndromes or acquired neurological conditions. Even within this mild NSID group, however, intellectual, cognitive–linguistic, and academic functioning is expected to vary along a continuum rather than forming sharply bounded subtypes, which has important implications for both assessment and classification.

While primarily psychometric and descriptive in nature, CHC theory also helps to structure the analysis of cognitive strengths and weaknesses in individuals with nonspecific intellectual disability. Prior work suggests that such individuals often show relatively preserved crystallised knowledge alongside more pronounced difficulties in working memory, auditory processing, processing speed, and learning efficiency (Lifshitz et al. 2018; Sajewicz-Radtke et al. 2025), and that phonological processing, phonological short-term memory, and rapid automatized naming are key predictors of decoding and reading fluency in students with mild intellectual disability of unspecified origin (Barker et al. 2013; Soltani and Roslan 2013). At the same time, CHC-based profiles provide limited insight into underlying neurodevelopmental mechanisms, learning dynamics, motivation, and the impact of educational environments, and they may be affected by floor effects as well as by insufficient coverage of pragmatic and interactional aspects of language that are critical for reading comprehension. Thus, in the present study, we use CHC as an organising and conceptual framework for selecting those broad abilities that are most strongly linked to literacy. Specifically, we focus on auditory processing (Ga), processing speed (Gs), and reading and writing (Grw). Other broad CHC abilities (e.g., visual processing, long-term memory, general knowledge) are not examined here, and our results should therefore not be interpreted as a comprehensive test of the full CHC model.

While the processes underlying reading development in typically developing children and those with specific reading disabilities are well-established (Castles and Coltheart 2004; Melby-Lervåg et al. 2012), research focusing on children with nonspecific intellectual disability remains scarce (Sermier Dessemontet and de Chambrier 2015). This limits our understanding of whether children with nonspecific intellectual disability follow broadly typical developmental trajectories, or whether they show more heterogeneous and less predictable patterns of reading acquisition than those reported in syndrome-specific groups.

A critical step is to identify specific reading and writing (Grw) profiles and key cognitive skills, including auditory processing (Ga; Melby-Lervåg et al. 2012), processing speed (Gs; Araújo et al. 2015), and oral language abilities (Nilsson et al. 2025), that influence literacy development in this group.

Reading acquisition in individuals with intellectual disability involves a complex interplay of strengths and weaknesses. While phonological decoding—establishing grapheme–phoneme correspondences—is foundational (Kirby et al. 2003; Parrila et al. 2004), individuals with intellectual disability often show marked deficits in this area compared to typically developing peers (Channell et al. 2013). These difficulties are compounded by weaknesses in phonological awareness and memory (Henry and Winfield 2010; Lemons and Fuchs 2010; Næss et al. 2012). In contrast, some studies suggest that orthographic processing and rapid automatised naming (RAN) skills in individuals with intellectual disability can be relatively better developed than phonological decoding, broadly aligning with their verbal abilities (Laing et al. 2001; Ypsilanti et al. 2006). RAN is generally viewed as a multi-component task involving visual recognition, rapid access to the phonological lexicon, retrieval of verbal labels, and speed of visuomotor integration, which together help explain its strong association with reading fluency. Additionally, recent findings emphasise the role of oral language skills as significant contributors to literacy outcomes (Nilsson et al. 2025).

Despite the growing evidence of literacy-related skills in individuals with intellectual disability, significant research gaps persist concerning those with nonspecific intellectual disability. While studies have often focused on mixed-aetiology samples or groups with specific syndromes (e.g., Down syndrome, Williams syndrome) (Abbeduto et al. 2007; Roch and Jarrold 2008; Verucci et al. 2006), targeted research on Grw, Ga, and Gs abilities in individuals with nonspecific intellectual disability is limited. This is crucial for determining whether the observed patterns are consistent across aetiologies or unique to particular syndromes.

### Aims of the Present Study

We conducted this exploratory study to address the gaps concerning reading and writing development and the role of cognitive variables such as auditory processing, processing speed, and oral language in individuals with nonspecific intellectual disability (Nilsson et al. 2025; Ratz and Lenhard 2013; Sermier Dessemontet and de Chambrier 2015; Sermier Dessemontet et al. 2017). Our primary research question was whether individuals with nonspecific intellectual disability exhibit distinct reading and writing, auditory processing, and rapid naming profiles. Identifying such patterns could provide valuable insights into the cognitive architecture of nonspecific intellectual disability and inform individualised intervention and placement decisions. Furthermore, we sought to examine whether our findings align with the ICD-11 classification, which posits that individuals with mild intellectual disability typically exhibit literacy skills commensurate with 3 to 4 years of primary education. Our study aimed to provide empirical data to inform this classification in the nonspecific intellectual disability group and to support the development of targeted literacy interventions. In addition to examining dimensional relations among the variables, we investigated whether individuals with NSID exhibit recurring multivariate performance patterns. This person-centred, exploratory component aimed to determine whether prototypical, probabilistic performance profiles emerge beyond general ability level, rather than representing exclusive categories, and whether such profiles correspond to educational placement and ICD-11 assumptions.

## 2. Materials and Methods

### 2.1. Participants

The study sample consisted of 122 individuals (72 boys and 50 girls) diagnosed with nonspecific intellectual disability, aged between 7 years and 6 months and 18 years and 7 months (*M* = 13.31, *SD* = 1.78), recruited from psychological consulting centres. The resulting age range was therefore broad, which is typical for school-based NSID samples but may increase heterogeneity in cognitive and academic performance. All participants had a clinical diagnosis of mild intellectual disability according to national guidelines and ICD-11 criteria and had no identified biomedical aetiology (e.g., known genetic syndrome, acquired brain injury), which is consistent with our use of the term nonspecific intellectual disability (NSID). Thus, the present sample represents the mild end of the NSID spectrum and does not include individuals with moderate or severe levels of intellectual disability. Inclusion criteria were (a) documented mild intellectual disability, (b) age between 7;6 and 18;7 years, (c) enrolment in a mainstream or special school, and (d) proficiency in the language of instruction as the native language. Exclusion criteria included (a) diagnosed moderate or severe intellectual disability, (b) known genetic or neurological conditions, and (c) uncorrected sensory impairments that would preclude valid assessment. Of the participants, 16% resided in small towns (less than 5000 inhabitants), 55% in medium-sized cities (up to 100,000), and 33% in large urban areas (more than 100,000). The majority were students in special education schools (61%), followed by public primary schools (35%). Regarding parental education, 70% of the mothers had primary or vocational education, 20% had completed secondary education, and 8% held a higher education degree (2% missing data). Of the fathers, the corresponding percentages were 79%, 7%, and 7%, respectively (7% missing data).

For clustering analysis, only those with complete data of all cognitive–linguistic variables (*n* = 110) were included. Additionally, a demographically matched comparison group (*n* = 110) was drawn from the Specialist Battery for the Diagnosis of Cognitive Abilities and School Skills (SB6/18) validation dataset (Jurek et al. 2024; Radtke and Sajewicz-Radtke 2024), consisting of individuals without any formal diagnosis. The matching criteria included age and sex. The final comparative dataset comprised 220 participants, with 50% representing individuals with mild nonspecific intellectual disability and 50% the control group.

### 2.2. Measures

Cognitive–linguistic processing skills were assessed using the SB6/18 (see Appendix A), a standardised instrument to evaluate core cognitive and school-related abilities in children and adolescents (Jurek et al. 2024; Radtke and Sajewicz-Radtke 2024). The battery includes the following seven subscales: (1) phonetic coding–linguistic aspect, (2) phonetic coding–cognitive complexity, (3) speed of perception, (4) verbal information processing speed, (5) decoding, (6) reading comprehension, and (7) writing. All scale scores were converted to a standardised 1–10 Standard Ten (STEN) scale using normative tables, which facilitates multivariate analyses without further standardisation.

Raw scores from five literacy-related tests administered across all age groups were analysed to compare the developmental age at which individuals with intellectual disability reached reading and writing performance levels with that of typically developing peers. These subtests included (1) pseudowords, (2) reading aloud, (3) true/false sentences, (4) sentences with gaps, and (5) words–sentences and written expression (see Appendix A).

### 2.3. Procedure

The data were collected individually by trained professionals in quiet, standardised conditions consistent with the guidelines of the SB6/18 battery. Informed consent was obtained from the legal guardians of the participants. The study was approved by the Ethics Committee for Research Projects at the Faculty of Social Sciences, University of Gdansk, Poland (decision no. 13/2022), and all procedures complied with the Declaration of Helsinki. In the second phase of the study, a demographically matched control sample was randomly selected from the SB6/18 normative database. Each individual with intellectual disability was paired with a control based on age and sex to enable direct group comparisons.

### 2.4. Analytical Strategy

The analytical strategy of this study encompassed three distinct components, each employing different statistical techniques and data subsets.

#### 2.4.1. Identification of Cognitive–Linguistic Processing Profiles Using Fuzzy Clustering

The first phase aimed to identify prototypical subgroups of individuals with intellectual disability based on their cognitive–linguistic processing patterns, rather than discrete or mutually exclusive categories. Although PCA indicated a strong first component, this variable-centred finding does not preclude exploring whether recurring profiles of strengths and weaknesses emerge at the individual level. To allow for flexible classification and to capture the fuzzy boundaries often present in psychological data, the fuzzy C-means (FCM) clustering algorithm was applied (Babuska 2001; Bezdek 1981; Dunn 1973). FCM offers a probabilistic assignment of individuals to clusters, which aligns with the inherent variability in cognitive and linguistic performance among individuals with intellectual disability. Accordingly, the resulting clusters should be interpreted as fuzzy, prototypical profiles that reflect degrees of similarity rather than strict membership. Models based on the FCM clustering were compared based on multiple clustering quality indices that provide complementary insights into model fit, compactness, and separation between clusters. The fuzzy silhouette index measures cluster coherence and separation in a fuzzy context, with higher values indicating a better fit. Partition entropy captures the degree of fuzziness in the partition, and lower values reflect more distinct clusters. The partition coefficient indicates the concentration of membership values, where higher values suggest clearer cluster assignment. The modified partition coefficient is a normalised version of the partition coefficient that adjusts for the number of clusters, and higher values are preferable. The Davies–Bouldin index assesses within-cluster compactness relative to between-cluster separation, with lower values denoting better clustering. Finally, the Calinski–Harabasz index evaluates the ratio of between-cluster to within-cluster variance, where higher values indicate more defined cluster structures.

As all variables were derived from the same psychometric battery and shared identical scales, standardisation was deemed unnecessary. To determine the optimal number of clusters, three methods implemented in the NbClust R package (3.0.1) were used: the elbow method, silhouette method, and gap statistics (Charrad et al. 2014). The fviz_nbclust() function facilitated visual determination of the optimal cluster solution. Cluster analysis was conducted using the fcm() function in the ppclust package. Random initialisations (k = 50) were used to mitigate the risk of local minima. This phase included data from 110 participants with complete responses on all seven variables. No imputation was conducted due to the small sample size and risk of bias due to the synthetic data.

#### 2.4.2. Comparative Analyses Using Matched Demographic Samples

The second phase compared the cognitive–linguistic processing skills between individuals with intellectual disability and a control group without any formal diagnosis. A demographically matched dataset of 220 observations (50% individuals with nonspecific intellectual disability and 50% controls) was constructed. Differences in the seven cognitive–linguistic skill domains were tested using Student’s t-tests with Holm–Bonferroni correction for multiple comparisons (Holm 1979).

#### 2.4.3. Estimating Developmental Age Equivalents for Reading and Writing Skills

The final and exploratory phase aimed to determine and compare the developmental age at which individuals with intellectual disability attain performance levels in reading and writing skills to those of typically developing individuals. Five literacy-related subtests that were administered across all age groups were analysed using local regression methods (LOESS) to model nonlinear patterns across age groups (Cleveland and Loader 1996). Developmental age equivalents were inferred by visually comparing raw score distributions of individuals with intellectual disability against the normative sample.

### 2.5. Data Preparation and Software

All data processing and statistical analyses were conducted using R (R Core Team 2025) in R (4.5.1). The following key packages were used: tidyverse (Wickham et al. 2019) for data manipulation; NbClust and factoextra for cluster evaluation (Charrad et al. 2014; Kassambara and Mundt 2020); ppclust for FCM clustering (Babuska 2001); and rstatix for statistical testing (Kassambara 2023).

## 3. Results

### 3.1. Cognitive–Linguistic Processing Profiles

**Figure 1:**
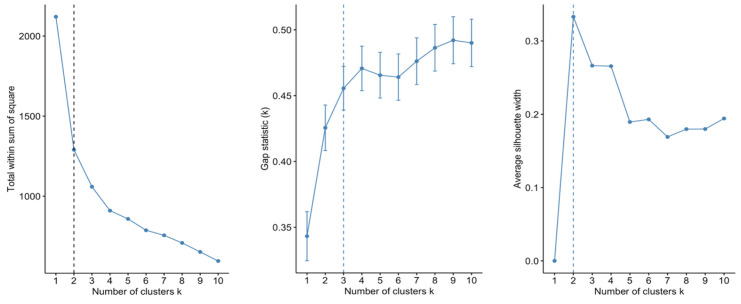
Results of the cluster number estimation using the elbow, silhouette, and gap statistic methods.

Both the elbow and silhouette methods indicated a two-cluster solution, whereas gap statistics suggested a three-cluster structure (Figure 1).

To assess the dimensionality of the dataset and guide visualisation, a principal component analysis (PCA) of the seven cognitive–linguistic variables was conducted. The PCA revealed that the first principal component accounted for 86.53% of the total variance, while the second explained an additional 5.07%. This indicated a predominantly unidimensional structure, with a dominant first component that can be interpreted as a general cognitive–linguistic performance index (or severity composite) derived from the seven subtests. Although PCA is a formative, variable-centred technique that constructs components as linear combinations of observed variables rather than estimating reflective latent factors, the high proportion of variance captured by the first component suggests that the subtests largely align along a single common dimension.

Next, two FCM clustering models were fitted: one specifying two and the other specifying three clusters. Table 1 shows the comparison between these models based on multiple clustering quality indices.

Based on these metrics, the two-cluster solution demonstrated superior model fit across all indicators. It yielded higher silhouette and partition coefficients, lower entropy, and more favourable Davies–Bouldin and Calinski–Harabasz indices. Furthermore, it was more easily interpretable within the framework of the theoretical assumptions underpinning the study.

**Figure 2:**
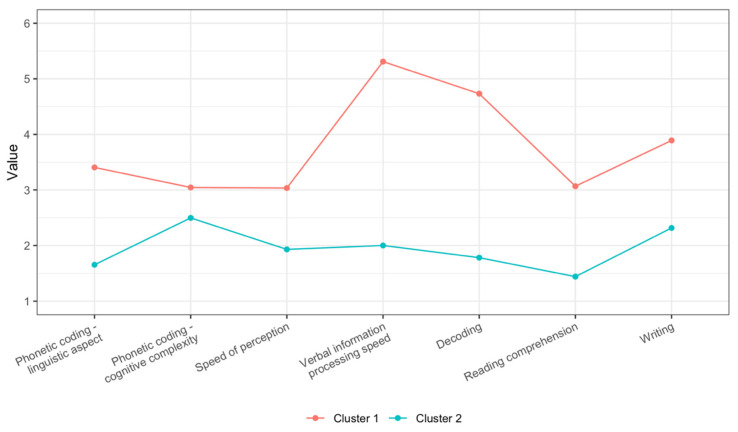
Cluster centroids for the 2-cluster fuzzy C-means solution across seven cognitive–linguistic processing skills.

The centroids for each cluster in the two-cluster model are presented in Figure 2. For comparison, the centroids for the three-cluster solution are included in Figure 1 in the Appendix B.

The two-cluster FCM solution revealed two prototypical cognitive–linguistic performance profiles in individuals with intellectual disability. Given the fuzzy nature of the classification and the strongly unidimensional structure indicated by the PCA, these profiles are best understood as level-differentiated performance patterns along the general cognitive–linguistic performance index, characterised by degrees of membership rather than mutually exclusive groupings. Cluster centroids (expressed as STEN scores ranging from 1 to 10) indicated notable differences in overall cognitive–linguistic performance levels. Profile 1 (Cluster 2, Figure 2: Globally Weaker Cognitive–Linguistic Skills, *n* = 64) was characterised by consistently low performance across all assessed domains. Individuals in this cluster exhibited particularly limited skills in phonetic coding–linguistic aspect, reading comprehension, and decoding. Other abilities were below the STEN scale midpoint, indicating a generalised cognitive–linguistic processing deficit. In contrast, individuals in Profile 2 (Cluster 1, Figure 2: Moderately Developed Cognitive–Linguistic Skills, *n* = 46) demonstrated stronger and more balanced performance across all domains. Key differentiators were as follows: verbal information processing speed was slightly above average, and decoding and writing approached normative levels. Results of this profile were more favourable than those of Profile 1 even in the lowest-scoring domain. This profile reflected individuals with moderately developed cognitive–linguistic competencies, who may exhibit greater functional independence in school or adaptive contexts.

Pearson’s Chi-squared test with Yates’ continuity correction revealed that sex was not significantly associated with cluster membership (χ^2^ = 0.27, *p* = 0.60). Similarly, independent-samples t-tests indicated no significant difference in mean age between students in the two clusters (*t* = 0.83, *p* = 0.41). However, a significant association was observed between school type and cluster membership, suggesting that cognitive–linguistic processing profiles varied by educational context (χ^2^ = 11.48, *p* < 0.01). Specifically, 70% of students attending special schools were assigned to Cluster 1, while the remaining 30% were in Cluster 2. In contrast, 61% of students attending regular or inclusive schools were classified into Cluster 2, with 39% assigned to Cluster 1.

### 3.2. Group Comparisons of Cognitive–Linguistic Processing Skills

**Figure 3:**
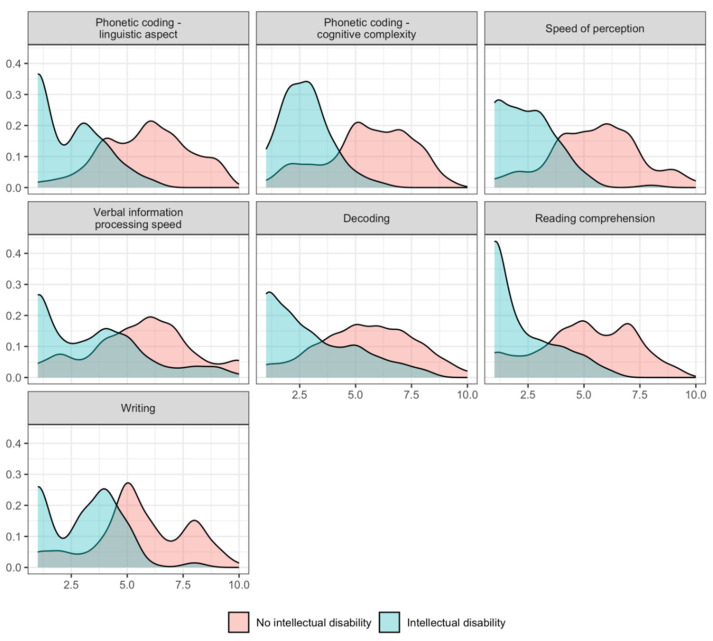
Density distributions of cognitive–linguistic processing skills in individuals with intellectual disabilities and the demographically matched control group.

Student’s t-tests with Holm–Bonferroni correction results are presented in Table 2, and the distributions of scores for both groups across all variables are shown in Figure 3.

All observed differences were statistically significant after correction, with large effect sizes across all variables. Students with intellectual disability consistently scored significantly lower than their demographically matched peers, particularly in phonetic coding–linguistic and speed of perception, where differences exceeded approximately two standard deviations. These findings underscore the pervasive nature of cognitive–linguistic processing challenges in individuals with mild intellectual disability.

### 3.3. Developmental Age Equivalents in Literacy-Related Skills

**Figure 4:**
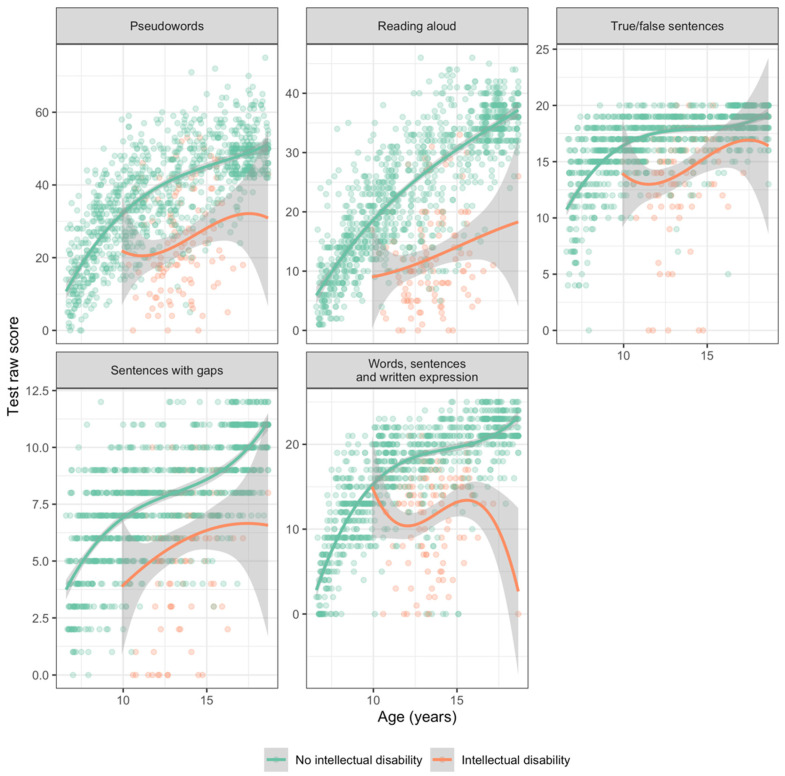
Developmental age equivalents in reading and writing subtests in students with intellectual disabilities, based on local regression method curves and normative comparisons.

The LOESS regression models showed smoothed trajectories of raw score development and enabled visual comparison with normative data (Figure 4). The highest model accuracy among students with intellectual disability was observed between the ages of 12 and 15. In contrast, estimates for younger and older participants were less precise, likely due to greater variability in raw scores.

Across all five subtests, students with intellectual disability consistently lagged behind their typically developing peers in skill acquisition, typically performing at levels observed in peers approximately 2–4 years younger during middle childhood and early adolescence. Performance tended to plateau after about age 15, with no further observable gains in average raw scores. Thus, their trajectories did not simply follow a parallel but delayed course; instead, they showed an earlier and lower plateau, suggesting that the attainable level of literacy skills was constrained. Because the LOESS approximation exhibited greater estimation uncertainty in participants above age 15, the exact shape and timing of this plateau should be interpreted with caution. This uncertainty reflects both increased variability in raw scores and the smaller number of older adolescents contributing to several subtests, which reduces the stability of the curve in the upper age range.

In pseudowords, students with intellectual disability, regardless of age, did not reach the average performance level of 10-year-olds from the normative sample. Ten- to twelve-year-olds with intellectual disability scored similar to typical 7-year-olds, and no improvement was observed beyond age 15.

In reading aloud, a nearly linear increase in raw scores was observed in students with intellectual disability, although at a slower developmental pace. At age 10, students with intellectual disability demonstrated reading abilities of typical 7-year-olds; by age 15, their performance approached that of 10-year-olds.

In true/false sentences, performance gains were modest. Students with intellectual disability aged 10–12 achieved scores typical for 7–8-year-olds, while 15-year-olds reached the level of 9-year-olds.

The sentences with gaps subtest showed a score distribution pattern similar in shape to the normative group but shifted by approximately 3–4 years. Ten-year-olds with intellectual disability performed at the level of typical six-year-olds, while fifteen-year-olds reached the level of nine-year-olds. Again, no meaningful progress was observed after age 15.

In words–sentences and written expression, students aged 10–15 with intellectual disability reached performance levels of 9–10-year-olds from the normative sample. However, in older students, a decline in performance with age was observed, possibly reflecting increasing cognitive or motivational difficulties with writing tasks.

## 4. Discussion

This study aimed to determine whether individuals with nonspecific intellectual disability exhibit specific reading and writing (Grw), auditory processing (Ga), and rapid naming profiles, and whether their developmental trajectories align with ICD-11 expectations for individuals with mild intellectual disability.

Using FCM clustering, we identified two prototypical cognitive–linguistic performance profiles in students with nonspecific intellectual disability, which primarily differed in overall level of functioning along a general cognitive–linguistic performance index suggested by the PCA. The first profile (Globally Weaker Cognitive–Linguistic Skills) exhibited profound and pervasive deficits across all assessed domains, particularly in phonetic coding, decoding, and reading comprehension. These findings are consistent with prior research results emphasising significant phonological decoding deficits in individuals with intellectual disability (Channell et al. 2013; Henry and Winfield 2010; Lemons and Fuchs 2010).

The second profile (Moderately Developed Cognitive–Linguistic Skills) revealed relatively better performance, especially in verbal information processing speed and writing skills, with decoding and writing approaching normative levels. This suggests that a subgroup of individuals with nonspecific intellectual disability may retain partial strengths in literacy-related areas, aligning with the view that some individuals with intellectual disability can exhibit relatively intact orthographic processing and RAN skills despite broader cognitive limitations (Laing et al. 2001; Ypsilanti et al. 2006).

From a methodological perspective, the PCA results showed that a single principal component accounted for over 86% of the variance, indicating that the seven cognitive–linguistic subtests largely shared a common dimension. Because PCA is a formative, variable-centred technique, this first component is best viewed as a composite index of general cognitive–linguistic performance rather than a reflective latent factor. This pattern suggests that the seven indicators primarily reflect a broad cognitive–linguistic severity dimension rather than multiple independent abilities. Accordingly, the two fuzzy clusters are best understood as level-of-functioning profiles along this general continuum—distinguishing students with globally weaker versus moderately developed cognitive–linguistic skills—rather than as qualitatively distinct cognitive subtypes. This interpretation is consistent with the use of fuzzy clustering, which assumes graded rather than sharply separated subgroups, and supports the use of these profiles as practically meaningful severity bands to inform educational decision-making.

Importantly, the profiles differed not by sex or age but by school type. Students from inclusive education settings were more likely to have the moderately developed profile, echoing evidence that enriched educational environments can support cognitive–linguistic development (Wei et al. 2011).

The LOESS analyses revealed that individuals with nonspecific intellectual disability reached literacy skill levels comparable to typically developing peers approximately 2–4 years younger during middle childhood and early adolescence. Importantly, their trajectories deviated from a simple delay pattern: performance plateaued after about age 15 at levels substantially below those of same-age peers, suggesting limited further gains rather than a delayed convergence. Critically, on average, students with mild nonspecific intellectual disability did not reach the expected literacy level of students who had completed 3–4 years of primary education even by late adolescence, contrary to the expectations of ICD-11. Taken together, these age-related patterns suggest that opportunities to further improve foundational literacy skills may be constrained once students with mild nonspecific intellectual disability enter secondary grades, potentially reflecting both limitations in underlying cognitive–linguistic resources and reduced instructional emphasis on basic reading and writing. These findings highlight the need for further research to better understand the developmental trajectories of reading and writing in this population.

For instance, in pseudoword decoding and sentence comprehension tasks—which rely heavily on phonological processing (Kirby et al. 2003; Parrila et al. 2004)—students with nonspecific intellectual disability could not reach the skill levels of 10-year-old typically developing children. These findings reinforce concerns raised by Ratz and Lenhard (2013) and Sermier Dessemontet and de Chambrier (2015) regarding persistent and marked reading difficulties in individuals with intellectual disability, particularly in decoding-related skills.

However, tasks involving writing and sentence-level comprehension showed comparatively better performance in older students, suggesting the gradual development of higher-level linguistic strategies, possibly compensating for phonological weaknesses (Laing et al. 2001).

### 4.1. Cognitive Underpinnings: CHC Model Perspective

Our results align with the CHC model’s emphasis on multiple, interrelated cognitive abilities, rather than a unitary IQ score, as a basis for understanding literacy outcomes (McGrew and Evans 2023; McGrew 2009). The significant deficits in phonological processing (Ga) and processing speed (Gs) observed in students with nonspecific intellectual disability highlight the role of these broad cognitive domains in literacy development (Araújo et al. 2015; Melby-Lervåg et al. 2012). The pronounced gap in phonetic coding skills and processing speed compared to controls underscores that interventions must extend beyond reading mechanics to address underlying cognitive deficits.

These findings support the ICD-11 approach of not relying solely on IQ scores for diagnosis but evaluating specific functional abilities such as literacy (Schneider and McGrew 2018). As shown in this study, reading ability in individuals with nonspecific intellectual disability cannot be fully predicted by their IQ level, corroborating prior claims (Share et al. 1989; Siegel 1989) that cognitive and reading skills are partially independent.

### 4.2. Implications for Practice

The two identified cognitive–linguistic profiles suggest the need for differentiated educational strategies. Students in the globally weaker profile may require intensive, phonologically based instruction tailored to their lower starting points, while those with the moderately developed profile could benefit from interventions that foster higher-level reading comprehension and writing skills.

Furthermore, interventions should target both phonological awareness and processing speed, as deficits in these domains critically impede literacy acquisition (Castles and Coltheart 2004; Melby-Lervåg et al. 2012). Specifically, interventions based on phonemic discrimination, RAN, and working memory training could offer benefits (Henry and Winfield 2010).

### 4.3. Limitations and Future Directions

While the present study provides important insights into cognitive–linguistic profiles in individuals with nonspecific intellectual disability, several limitations must be acknowledged.

First, because of the cross-sectional design, the age-related patterns captured by the LOESS curves should be interpreted as apparent developmental trajectories rather than as direct evidence of intra-individual change. In particular, the plateau and apparent decline in reading and writing performance after 15 years of age cannot be taken as conclusive evidence of cognitive regression; they may instead reflect motivational factors, educational limitations, or other external influences. Future longitudinal studies are needed to track literacy development into young adulthood and beyond, to clarify which of these explanations is most plausible and to illuminate the mechanisms underlying these late-stage developmental trends (Sermier Dessemontet and de Chambrier 2015; Wei et al. 2011).

Second, although the study identified clear differences in literacy-related profiles depending on school type (special vs. inclusive), the nature of this relationship remains ambiguous. Students in special schools may be systematically lower-performing due to more severe cognitive and linguistic impairments. However, alternative explanations such as differences in school resources, instructional methods, or expectations may exist. Future research should directly examine the cognitive and linguistic profiles of students within different educational settings, ideally controlling for baseline intellectual and language abilities, to disentangle student-related versus context-related effects (Allor et al. 2014; Koritsas and Iacono 2011).

Third, the study revealed that students in Profile 1 (Globally Weaker Cognitive–Linguistic Skills) were disproportionately enrolled in special education settings. This raises the question of whether these students exhibit both general intellectual disability and developmental learning disorder (DLD) with impairment in reading, akin to profiles observed in developmental dyslexia (Channell et al. 2013; Roch and Jarrold 2008). Although ICD-11 recommends caution when diagnosing DLD in individuals with intellectual disability—given that academic limitations are often a natural consequence of generalised cognitive deficits—it acknowledges that this diagnosis is possible when learning difficulties are significantly greater than that expected based on the individual’s overall intellectual functioning. Therefore, targeted research on reading abilities in individuals with nonspecific intellectual disability is essential to better distinguish between general developmental delays and specific reading disorders. Future investigations should systematically assess the prevalence and characteristics of comorbid reading disabilities, using both global cognitive evaluation and specific diagnostic criteria for DLD, to ensure that students receive appropriately individualised support and interventions.

Fourth, although we used age-standardised STEN scores to mitigate age-related mean differences, the wide age range (7–18 years) may still have contributed to shared age-related variance across measures, potentially accentuating the prominence of a general factor in the PCA. Future studies using narrower age bands or longitudinal designs would allow a more precise separation of age effects from underlying cognitive structure.

Fifth, another limitation concerns the developmental analyses. Age-equivalent estimates were based on visual inspection of LOESS curves without statistical confidence intervals or quantitative interpolation of normative crossing points. Combined with the smaller number of older adolescents in the dataset, this limits the precision of conclusions about the late-adolescent plateau or decline. Future studies should apply more rigorous modelling approaches to clarify developmental change during this period.

Sixth, although fuzzy C-means clustering produces membership coefficients that quantify the degree to which each individual belongs to each profile, we did not present a detailed analysis of these membership values in the current paper. As a result, the sharpness of the separation between the two prototypical profiles cannot be fully evaluated, and the clusters should be interpreted as broad, level-based performance patterns on a general cognitive–linguistic continuum rather than sharply differentiated subtypes. Future studies with larger samples and a more specific focus on person-centred modelling should analyse the full distribution of membership degrees and, where appropriate, combine dimensional and mixture approaches to further clarify the psychological meaning and robustness of the profiles.

Finally, although the sample was relatively large and well-characterised, it belonged to a single national educational and cultural context, which may restrict generalisability. The patterns observed here therefore primarily apply to students with mild NSID educated within this specific system and may not generalise to other countries with different curricula, support structures, or placement practices. In line with ICD-11, functional academic skills in reading and writing are primarily expected in individuals with mild disorders of intellectual development, whereas conventional academic reading is typically unattainable for individuals with severe intellectual disability. Accordingly, future research should prioritise cross-cultural replication of the present findings in students with mild NSID, particularly in languages with different orthographic and morphological properties, rather than extending this literacy framework to more severe levels of intellectual disability (Di Blasi et al. 2018; Ratz and Lenhard 2013).

The following are the scopes of future research:

- Employing longitudinal designs to investigate developmental literacy trajectories and potential late-adolescent declines.
- Examining the interplay between students’ cognitive profiles and educational context (e.g., school type, instructional methods).
- Assessing comorbid reading disabilities in individuals with weaker cognitive–linguistic profiles to refine intervention strategies.
- Expanding samples of students with mild NSID across diverse cultural and educational contexts to enhance the generalisability of findings.

This is essential to build a more nuanced and developmentally informed understanding of literacy in individuals with nonspecific intellectual disability, ultimately guiding the development of tailored, effective educational interventions.

## 5. Conclusions

This study provides novel insights into the cognitive–linguistic diversity and literacy development of students with nonspecific intellectual disability. By applying the Carroll–Horn–Cattell framework and fuzzy C-means clustering, we identified two prototypical level-differentiated cognitive–linguistic profiles, highlighting substantial heterogeneity in processing abilities within this population. Literacy skills in students with NSID were significantly delayed compared to their typically developing peers and appeared to plateau by mid-adolescence, failing to meet the ICD-11 (WHO 2022) expectation of achieving literacy equivalent to 3–4 years of primary education.

These findings underscore the importance of shifting from a unitary IQ-based diagnostic and educational model to one that recognises specific cognitive–linguistic strengths and weaknesses. The results support the relevance of CHC-informed, multivariate assessment frameworks that consider several literacy-related and cognitive–linguistic abilities, and suggest that targeted, profile-informed literacy interventions are essential for this group. Moreover, this study provides empirical support for refining educational expectations and practices for individuals with mild NSID and contributes to more accurate and functional classifications in both clinical and educational contexts.

## Figures and Tables

**Table 1:** Selected fit indices for the 2-cluster and 3-cluster fuzzy C-means models.

Fit Measure	2-Cluster Model	3-Cluster Model
Fuzzy silhouette index	0.64	0.54
Partition entropy	0.49	0.82
Partition coefficient	0.68	0.52
Modified partition coefficient	0.36	0.28
Davies–Bouldin index	1.25	1.63
Calinski–Harabasz index	69.00	53.43

**Table 2:** Comparison of cognitive–linguistic processing skills between students with intellectual disability and those without diagnosis.

Cognitive–Linguistic Processing Skills	Individuals with ID Group (M ± SD)	Control Group (M ± SD)	T	Adj. *p*-Value	Cohen’s d	
Phonetic coding–linguistic aspect	2.37 ± 1.46	5.84 ± 1.92	15.05	<0.01	2.03	Large
Phonetic coding–cognitive complexity	2.73 ± 1.09	5.54 ± 1.93	13.27	<0.01	1.79	Large
Speed of perception	2.41 ± 1.30	5.51 ± 1.97	13.77	<0.01	1.86	Large
Verbal information processing speed	3.43 ± 2.36	5.58 ± 2.28	6.89	<0.01	0.93	Large
Decoding	2.99 ± 2.00	5.45 ± 2.14	8.82	<0.01	1.19	Large
Reading comprehension	2.12 ± 1.46	4.99 ± 2.16	11.54	<0.01	1.56	Large
Writing	2.99 ± 1.66	5.55 ± 2.13	9.93	<0.01	1.34	Large

**Figure A1:**
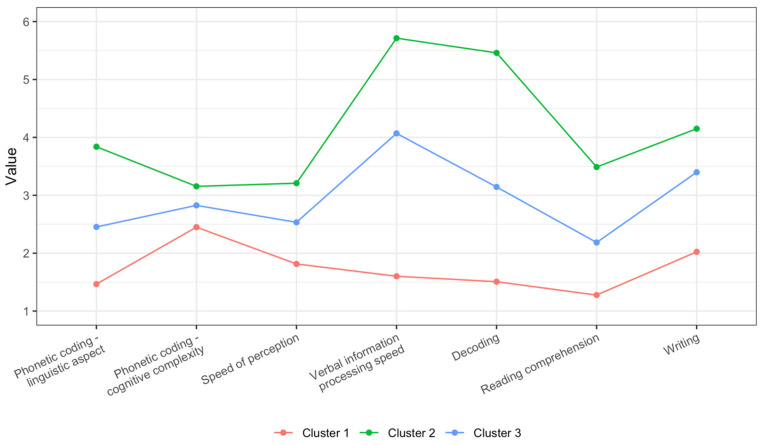
Cluster centroids for the 3-cluster fuzzy C-means solution across seven cognitive–linguistic processing skills.

## Data Availability

The original data presented in the study are openly available in the Open Science Framework (OSF) at https://osf.io/e4smx/?view_only=366e50ebafec4493b95756db55170dd1 (accessed on 19 November 2025).

## Abbreviations

The following abbreviations are used in this manuscript:

CHC	Carroll–Horn–Cattell
DLD	developmental learning disorder
FCM	fuzzy C-means clustering
Ga	auditory processing
Grw	reading and writing skills
Gs	processing speed
ICD-11	International Classification of Diseases 11th Revision
ID	intellectual disability
IQ	intelligence quotient
LOESS	local regression methods
NSID	nonspecific intellectual disability
PCA	principal component analysis
RAN	rapid automatised naming
SB6/18	Specialist Battery for the Diagnosis of Cognitive Abilities and School Skills

## Appendix A

(1) Structure of the Specialist Battery for the Diagnosis of Cognitive Abilities and School Skills (SB6/18) Diagnostic Tool

The Specialist Battery for the Diagnosis of Cognitive Abilities and School Skills (SB6/18) is a standardised psychometric tool to assess a wide range of cognitive processes and academic skills essential for learning. The structure of the battery is based on the Cattell–Horn–Carroll (CHC) theory of cognitive abilities, integrating both broad and narrow cognitive domains.

The tool consists of six core scales, each targeting a distinct area of functioning: visual–spatial processing, auditory–linguistic processing, processing speed, long-term memory, knowledge, and reading and writing (Table A1). Each scale is divided into subscales that correspond to specific processes or skill sets, which are evaluated through one or more psychometric tests or indicators.

Table A2 presents a summary of the main cognitive functions and skills assessed within each of these six scales. Table A3 presents an overview of the SB6/18 tests and indicators, alongside the specific cognitive or academic functions assessed by the tests. Finally, Table A4 briefly describes all 27 individual tests included in the battery. These descriptions highlight the nature of the task and the type of performance assessed, offering practical insights into how the battery captures key learning-related abilities.

Together, these tables provide a comprehensive overview of the SB6/18’s theoretical and operational framework, making it a valuable appendix for researchers and practitioners in educational and psychological assessment contexts. Table A5 provides an overview of the psychometric properties of each SB6/18 test, including reliability coefficients and evidence of validity.

This formatting facilitates the identification of relevant components used for analysis and interpretation in the study’s context.

**Table A1:** Overview of scales, subscales, and tests of SB6/18.

Scale	Subscale	Test/Indicator
I. Visual–spatial processing	Visualisation	Visual analysis and synthesis
	Rotation speed	Figure rotation
	Perceptual organisation	Gdańsk figure—copy
	Visual memory	Gdańsk figure—memory
	Visual–motor coordination	Gdańsk figure—coordination indicators
II. Auditory–linguistic processing	Phonetic coding–linguistic aspect	Syllabic and phonemic analysis and synthesis
		Paronym analysis
	Phonetic coding–cognitive complexity	Syllabic inversion and spoonerisms
	Resistance to auditory distractors	Auditory distractors
III. Processing speed	Perceptual speed	Symbols, pair crossing, rapid naming
	Verbal processing speed	True/false sentences—time
IV. Long-term memory	Associative memory	Associations
	Semantic memory	Recall
V. Knowledge	General knowledge	Where? What?
	Lexical knowledge	Synonyms, antonyms
VI. Reading and writing	Decoding	Pseudowords
	Reading comprehension	Letter recognition, reading aloud, true/false sentences, gap sentences, new planet
	Writing	Words, sentences, and written statement
	Orthography	Dictation

**Table A2:** Functions and skills assessed by each SB6/18 scale.

Scale	Assessed Function or Skill
Visual–spatial processing	Cognitive abilities related to processing and interpreting visual–spatial information, including shapes, sizes, orientation, and spatial relationships.
Auditory–linguistic processing	Abilities to differentiate, memorise, and process auditory stimuli such as phonemes and syllables and to comprehend and interpret verbal information.
Processing speed	Speed and automaticity in executing repetitive, simple tasks under attentional control.
Long-term memory	Ability to acquire, store, and consolidate new information over time.
Knowledge	Scope of general and lexical knowledge acquired through experience and education.
Reading and writing	Basic school skills required for continued education and independent functioning in the world.

**Table A3:** Tests and indicators of SB6/18 and the assessed functions. In the table, the elements included in the present study have been highlighted in bold.

Test/Indicator	Assessed Function or Skill
**Rapid naming**	Speed of visual information processing and lexical access.
**True/false sentences—time**	Verbal information processing speed.
Associations	Ability to memorise and link information through associations.
Recall	Ability to retrieve information from memory after a single hearing.
Where?/what?	Scope of general knowledge.
Synonyms/antonyms	Lexical knowledge.
**Pseudowords**	Phonological decoding.
**Letter recognition**	Letter recognition skills.
**Reading aloud**	Effectiveness of reading aloud with comprehension.
**True/false sentences**	Reading comprehension and logical reasoning.
**Gaps in sentences**/new planet	Semantic comprehension and silent reading comprehension.
**Written words, sentences**	Writing competence.
Dictation	Spelling and short-term memory.

**Table A4:** Description of tests and indicators of SB6/18. In the table, the elements included in the present study have been highlighted in bold.

Test/Indicator	Description
**Pseudowords**	Participants read aloud a list of pseudowords with increasing phonological complexity in one minute. The number of accurately read pseudowords served as a measure of phonological decoding efficiency.
**Letter recognition**	Name the letters indicated by the diagnostician.
**Reading aloud**	Children read short sentences aloud and decide whether each sentence is true or false, providing a measure of oral reading with basic sentence-level comprehension rather than isolated word reading.
**True/false sentences**	Read the sentences silently and simultaneously assess their meaning (true or false).
**Sentences with gaps**	Silently read the sentences containing gaps and mark the words that best complete them (to choose from 3).
New planet	Silently read a short text of the story and then answer specific questions about its content.
Dictation	Write down the text dictated to them correctly in terms of spelling and punctuation.
**Syllable and phoneme analysis and synthesis**	Segment words into individual syllables or phonemes (analysis) and combine individual sounds or syllables to form complete words (synthesis).
**Analysis of paronyms**	Determine whether two presented words are identical or different. If the words are different, the participants must specify which phonemes differentiate them.
**Syllable inversion and spoonerisms**	Swap the positions of syllables in two-syllable words, while the “spoonerisms” part requires them to exchange the initial syllables of two-word phrases.
Auditory distractors	Answer questions presented through headphones, which are obscured by white noise and randomly spoken syllables.
Symbols	Fill in designated symbols in blank spaces beneath illustrations, with each drawing corresponding to a specific symbol.
Crossing out pairs	Identify and cross out each instance of the specified pair “cat–clock” from a series of stimuli presented in a specific order.
Quick naming	Quickly name represented objects.
**True/false sentences—time**	The time taken by the participant to complete the “true/false sentences” task.
Visual analysis and synthesis	Analyse and synthesise visual stimuli, reconstructing shapes and visual information.
Figure rotation	Mentally rotate geometric figures to determine matching shapes.
Gdańsk figure—copy	Copy a complex figure by analysing its visual structure.
Gdańsk figure—memory	Recall and reproduce a previously shown visual figure.
Gdańsk figure—coordination	Perform tasks requiring visual–motor coordination, combining hand movements with visual input.
Associations	Memorise pairs of items and later recall the associated element when prompted.
Recall	Hear short narratives and recall them from memory.
Where?	Answer general knowledge questions about spatial or factual concepts.
What?	Answer questions probing common knowledge and vocabulary.
Synonyms	Identify words with similar meanings from a list of options.
Antonyms	Identify words with opposite meanings from a list of options.
**Words, sentences, and written statement**	Write the given words and sentences and create a short written statement.

(2) Psychometric Properties of Specialist Battery for the Diagnosis of Cognitive Abilities and School Skills (SB6/18)

The psychometric analysis of the SB6/18 battery demonstrates excellent measurement properties across its scales, subscales, and individual tests. Internal consistency, as assessed by Cronbach’s alpha, ranged from α = 0.85 to α = 0.92 across subtests, indicating high reliability for both cognitive and academic skill measures. Additionally, the overall reliability coefficient for the cognitive and academic skills quotient was remarkably high (α = 0.96), suggesting that the battery provides a stable and consistent evaluation of learning-relevant cognitive processes.

Test–retest reliability estimates for a selected group of participants ranged from *r* = 0.84 to 0.90, supporting the temporal stability of SB6/18 outcomes. The confirmatory factor analysis (CFA) provided strong evidence of the battery’s structural validity, with fit indices indicating an excellent model fit (root mean square error of approximation, RMSEA = 0.045, comparative fit index, CFI = 0.92). These results confirm that the assumed hierarchical structure—aligned with the CHC theory of cognitive abilities—is well represented in the data.

Evidence for convergent validity was observed through strong correlations with other established measures of cognitive functioning, reading, writing, and academic achievement. Specific subscales (e.g., auditory–linguistic processing, visual–spatial processing, reading and writing) showed expected relationships with external criteria, supporting the battery’s theoretical foundations and applied diagnostic utility.

Overall, the SB6/18 battery can be considered a psychometrically sound tool for the comprehensive assessment of cognitive processes and academic skills in children and adolescents aged 6 to 18 years. Its high reliability, confirmed factorial validity, and robust external validity make it suitable for both clinical and research applications focused on learning difficulties and educational planning.

**Table A5:** Psychometric properties of the SB6/18 battery.

Scale	Subscale	Test/Indicator	Reliability (Cronbach’s α/Test–Retest)	Validity
Visual–spatial processing	Visualisation	Visual analysis and synthesis	α = 0.91	Strong structural agreement, correlations with TONI-4 *
Visual–spatial processing	Rotation speed	Figure rotations	α = 0.88	Correlations with fluid intelligence tests
Visual–spatial processing	Perceptual organisation, visual memory	Gdańsk figure—copy, memory	α = 0.89	Confirmed validity based on structural model
Visual–spatial processing	Visuomotor coordination	Gdańsk figure—coordination indicators	α = 0.86	Correlations with psychomotor coordination tests
Auditory–linguistic processing	Phonetic coding–linguistic aspect	Syllabic and phonemic analysis, paronyms	α = 0.92	Strong relations with speech and reading comprehension
Auditory–linguistic processing	Phonetic coding–cognitive complexity	Syllable inversion and spoonerisms	α = 0.89	Associations with decoding skills
Auditory–linguistic processing	Resistance to auditory distractors	Auditory distractors	α = 0.86	Confirmed construct validity
Processing speed	Perceptual speed	Symbols, pair cancellation	α = 0.90	Strong correlations with information processing speed
Processing speed	Verbal information processing speed	True/false sentences—time	α = 0.85	Consistency with SB5 ** outcomes
Long-term memory	Associative memory	Associations	α = 0.88	Strong relations with TOMAL-2 ***
Long-term memory	Semantic memory	Recall	α = 0.89	Consistency with semantic memory performance
Knowledge	General knowledge	Where? What?	α = 0.87	Correlations with crystallised knowledge
Knowledge	Lexical knowledge	Synonyms, antonyms	α = 0.88	Links with vocabulary breadth
Reading and writing	Decoding	Pseudowords	α = 0.90	Validity regarding reading difficulties
Reading and writing	Comprehension	Letter recognition, oral reading, true/false sentences, Cloze sentences, new planet	α = 0.91	Correlations with text comprehension and phonology
Reading and writing	Writing	Words, sentences, and written expression	α = 0.87	Validity regarding writing ability
Reading and writing	Orthography	Dictation	α = 0.88	Strong links with dysorthographia diagnosis

* TONI-4—Brown, L., Sherbenou, R. J., Johnsen, S. K. (2010). *Test of nonverbal intelligence* (4th ed.). Austin, TX: PRO-ED. ** SB5—Roid, G. H., Sajewicz-Radtke, U., Radtke, B. M. & Lipowska, M. *Skale Inteligencji Stanford-Binet*, *Edycja Piąta* [Stanford-Binet Intelligence Scales, Fifth Edition]. Gdańsk: Pracownia Testów Psychologicznych i Pedagogicznych [Laboratory of Psychological and Pedagogical Tests] (2017). *** TOMAL-2—Reynolds, C. R., Voress, J. K., Sajewicz-Radtke, U., & Radtke, B. M. (2024). *Test Pamięci i Uczenia się TOMAL-2* [Test of Memory and Learning TOMAL-2]. PracowaniaTestów Psychologicznych i Pedagogicznych [Laboratory of Psychological and Educational Tests].

General reliability for the entire SB6/18 battery (cognitive and academic skills quotient) was α = 0.96. Overall validity was confirmed through CFA with the following fit indices: root mean square error of approximation (RMSEA) = 0.045 and comparative fit index (CFI) = 0.92. Test–retest reliability for selected subtests ranged from r = 0.84 to 0.90.
